# Vaccines Directed Against Microorganisms or Their Products Present During Biofilm Lifestyle: Can We Make a Translation as a Broad Biological Model to Tuberculosis?

**DOI:** 10.3389/fmicb.2016.00014

**Published:** 2016-01-21

**Authors:** Mario A. Flores-Valdez

**Affiliations:** Centro de Investigación y Asistencia en Tecnología y Diseño del Estado de Jalisco, A.C. Biotecnología Médica y FarmaceúticaGuadalajara, Mexico

**Keywords:** vaccines, tuberculosis, biofilms, animal models, natural hosts

## Abstract

Tuberculosis (TB) remains as a global public health problem. In recent years, experimental evidence suggesting the relevance of *in vitro* pellicle (a type of biofilm formed at the air-liquid interface) production as a phenotype mimicking aspects found by *Mycobacterium tuberculosis*-complex bacteria during *in vivo* infection has started to accumulate. There are still opportunities for better diagnostic tools, therapeutic molecules as well as new vaccine candidates to assist in TB control programs worldwide and particularly in less developed nations. Regarding vaccines, despite the availability of a live, attenuated strain (*Mycobacterium bovis* BCG) since almost a century ago, its variable efficacy and lack of protection against pulmonary and latent disease has prompted basic and applied research leading to preclinical and clinical evaluation of up to 15 new candidates. In this work, I present examples of vaccines based on whole cells grown as biofilms, or specific proteins expressed under such condition, and the effect they have shown in relevant animal models or directly in the natural host. I also discuss why it might be worthwhile to explore these approaches, for constructing and developing new vaccine candidates for testing their efficacy against TB.

## Introduction

Since its introduction in 1921, *Mycobacterium bovis* BCG has been used to immunize around three billion people worldwide, with close to 115 million new doses applied each year (Andersen and Doherty, 2005; Skeiky and Sadoff, 2006), which have proven to be effective in protecting against severe, disseminated forms of tuberculosis (TB). Despite this success, opportunities to improve this vaccine are still evident, as this does not protect against the establishment of pulmonary infection nor latent disease. Bacteria living within a structure comprising metabolically and phenotypically diverse cells, covered by an extracellular matrix are often termed biofilms and compared to free-living (planktonic) cells have been shown to be more tolerant to drug treatment, induce different immune response and persist longer within infected tissues *in vivo* (Ojha et al., 2008). Considering that tuberculosis is characterized for chronicity of infection, as well as the known need of prolonged multiple antibiotic treatment to manage TB, even in drug-susceptible cases, it has been proposed that mycobacterial biofilms, where bacteria grow to produce a thick aggregate of mycolic acids at the air-liquid interface and exhibit increased phenotypic resistance to antibiotics (Ojha et al., 2008), might mimic aspects found during *in vivo* infection (Flores-Valdez et al., 2015), and that perhaps drug-persisters found in Guinea pigs might constitute some sort of biofilm (Orme, 2014).

Here, I present evidence of how using bacteria adapted to grow as biofilms or components derived from it, constitute effective preventive measures against some infectious diseases, either in relevant animal models or within the natural host. These were obtained from Pubmed using “biofilm vaccine” as keyword (295 records published on November 5, 2015), and selecting only original research papers showing evidence of evaluation of efficacy vs. *in vivo* challenge. Finally, I propose the use of a similar strategy to develop new TB vaccine candidates.

## Vaccines based on whole cells with altered capacity to produce biofilms

*Staphyloccocus aureus* is a Gram-positive pathogen well-known to produce biofilms, which are relevant in medical-device associated infections in humans, as well as in mastitis produced in animals. Using *in vitro* subculture of weak biofilm producer strains to obtain strong biofilm producer bacteria, it was found that the latter strains were more able to induce antibodies vs. poly-N-acetyl-β-1,6-glucosamine (PNAG), which ultimately led to better control of mastitis in sheep (Perez et al., 2009). Perhaps PNAG production was not the only factor altered during adaptation from weak to strong biofilm production, leading to better control of infection, however this study showed the use of biofilm-grown *S. aureus* to protect against infection. Similarly, *S. aureus* cultured as biofilm improved protection vs. mastitis in a mice model (Gogoi-Tiwari et al., 2015) compared to planktonic cells.

On the other hand, a UDP-glucose dehydrogenase deletion mutant of *Edwardsiella tarda*, showed increased autoaggregation and biofilm production, reduced survival in macrophages and reduced LD_50_ with increased survival in zebrafish, along with a dose-dependent protection of turbot vs. a lethal challenge (Lv et al., 2012). For another fish pathogen, *Aeromonas hydrophila*, incorporation of heat-inactivated, biofilm-cultured bacteria into the diet of catfish, promoted survival of 90–100% of vaccinated and then infected fish vs. a 30–40% protection conferred by a vaccine comprised of planktonic cells (Nayak et al., 2004), where improvement was suggested to depend on a modification of LPS in biofilm-grown cells (Asha et al., 2004), although the effect of changes found in proteins on vaccine efficacy was not evaluated in either study. Similar improved protection of biofilm-derived vaccine was also found against *A. hydrophila* for the snakehead murrel (*Chana striata*; Siriyappagouder et al., 2014).

Taken together, these studies demonstrate that there is enhanced protection against virulence challenge for several pathogens in their natural hosts or relevant animal models when biofilm-grown cells are used as vaccines.

## Vaccines based on mixed components obtained from biofilms

Another source of vaccine candidates are particular fractions obtained from cells or culture filtrates or a mixture of variable complexity composed of secreted proteins. In this regard, the utilization of extracellular proteins found within the biofilm matrix of *S. aureus* reduced the number of bacterial cells found inside a biofilm and surrounding tissue in a model of mesh-associated infection, as well as limited organ colonization upon dispersion of pathogen from biofilm (Gil et al., 2014).

In a somewhat opposed manner, *Bordetella pertussis* biofilm-derived membrane proteins protected against lung colonization in mice, although at levels lower than those produced by acellular pertussis vaccine currently in use, which is derived from planktonic cultures (de Gouw et al., 2014). It would be interesting to test particular components of each vaccine to compare their efficacy, or add biofilm-exclusive antigens to vaccines already in use to see if they can improve long-term protection, which was the aim of analyzing biofilm extracts in the study by de Gouw et al. (2014).

## Vaccines based on specific proteins produced during biofilm growth

When specific antigens show the capacity to induce a protective immune response, they can be used as subunit vaccines, employed in order to reduce harmful side effects produced by whole-cell products. A recombinant form of Biofilm associated protein (Bap) from *Acinetobacter baumannii* was able to induce production of specific antibodies, to reduce bacterial replication in liver and spleen of mice infected intraperitonialy, which succumbed 24 h post-infection when not immunized (Fattahian et al., 2011). Moreover, in another study, the use of recombinant Bap in combination with either Outer Membrane Vesicles derived from an *A. baumannii* strain devoid of 3-O-linked acyl chain from the disaccharide backbone of lipid A, or Outer Membrane Protein A, reduced bacterial replication in spleen and protected against death of infected mice (Badmasti et al., 2015).

On the other hand, a fragment obtained from *S. epidermidis* surface exposed protein C (SseC) when used as subunit vaccine, induced an immune response that inhibited biofilm formation in a foreign body infection model developed in rats (Shahrooei et al., 2012). Another *S. epidermidis* recombinant antigen, derived from Accumulation associated protein (Aap), induced production of antibodies capable of inhibiting biofilm formation *in vitro*, and led to a better control of an implant-originated infection in mice compared to ovalbumin-vaccinated groups (Yan et al., 2014). Furthermore, the Major amidase (Atl-AM, a multi-functional non-covalently cell wall associated protein involved in biofilm formation) from *Staphylococcus* induced both Th1 and Th2 response, increased opsonophagocytic killing of *S. aureus ex vivo*, reduced pathogen replication in heart, liver, and kidney and ultimately reduced death of infected mice compared to mock-immunized controls (Nair et al., 2015). All these evidences support the idea that specific components that are relevant for biofilm production, as vaccine candidates, which merits further research to determine their safety profiles and efficacy in other models or the actual host.

## Mycobacterial biofilms and the quest for new vaccine candidates against tuberculosis

The relevance of biofilms produced by pathogenic mycobacteria has recently regained attention, particularly for drug tolerance (Ojha et al., 2008; Ackart et al., 2014a), where it has been suggested that persisters found after drug treatment *in vivo* might resemble some sort of biofilm (Orme, 2014), and the possible role of this structure during infection has already been discussed (Flores Valdez et al., 2014), and it is summarized in Table 1. I do not mean to say that biofilm must indeed exist during *in vivo* infection, only that this mode of growth resemble to some extent aspects found by *Mycobacterium tuberculosis* during interaction with its host.

**Table 1:** **Key recent findings associating biofilms produced by *M. tuberculosis*-complex bacteria with relevant phenotypes *in vitro* and *in vivo***.

**Finding**	**References**
*M. tuberculosis* tolerates more antibiotics *in vitro* within biofilms than as planktonic cells	Ojha et al., 2008
*M. bovis* Δ*glnA1*produces less biofilms, is more susceptible to drugs and is less virulent in BALB/c mice compared to Wt bacteria	Chandra et al., 2010
TCA1, a small molecule that inhibits *M. tuberculosis* biofilms *in vitro*, reduces bacillary burden in lungs of infected BALB/c mice	Wang et al., 2013
Guinea pigs infected with *M. tuberculosis* produce antibodies against specific proteins present in *in vitro* grown biofilms	Kerns et al., 2014
2-aminoimidazole derivatives that disrupt *in vitro* grown *M. tuberculosis* biofilms restore susceptibility to drugs	Ackart et al., 2014b
*M. tuberculosis* Δ*mmaA4* produces less biofilms and is more susceptible to rifampicin *in vitro*	Sambandan et al., 2013
Leukocyte extracts favors *M. tuberculosis* biofilm production and drug tolerance *ex vivo*	Ackart et al., 2014a
*M. bovis* BCGΔ*BCG1419c* produces more biofilm *in vitro* and persists more in lungs and spleens of BALB/c mice	Flores-Valdez et al., 2015

Most manufacturers grow BCG for vaccine administration to humans, as a surface pellicle in liquid Sauton medium (Eickhoff, 1977). We have shown that Sauton medium favors formation of pellicle compared to Middlebrook 7H9 (Flores-Valdez et al., 2015), and others already demonstrated that pellicle mode of growth in Sauton medium render BCG more able to persist within macrophages, induce stronger inflammatory response but ultimately resulted in less control of bacterial replication in lungs of aerosol infected C57BL/six mice, after 3 months of infection with low dose *M. tuberculosis* H37Rv (Venkataswamy et al., 2012). Based on these results, it seems rather logical to think that biofilm mode of growth has not proven to be a useful source of antigens to protect against pulmonary and latent TB. Conversely, it suggests it would be worth shifting BCG vaccine production to Middlebrook 7H9 medium in the presence of detergent to obtain individual, planktonic cells.

Nevertheless, I think that such a failure to induce protective immunity by current BCG vaccines produced as biofilms might be at least partially circumvented if we were able to enhance biofilm production by BCG or by attenuated strains of *M. tuberculosis*. We should definitely first define the key molecular contributors to biofilm production by mycobacteria with their expression during *in vivo* infection. Perhaps current growth conditions has led to a lack of or suboptimal expression of relevant antigens from “weak” biofilm-producer BCG strains, or that under such conditions BCG maintains antigens that induce very strong but not protective immune response. This could be linked to a reduced capacity to remain within the host (or an enhanced clearance due to immune response early after vaccination), which consequently reduces the opportunity to express relevant antigens for all stages of infection.

In term of the capacity to remain within the host, we have recently shown that pellicle production and persistence in immunocompetent mice are linked in BCG (Flores-Valdez et al., 2015), and there is evidence that a BCG strain more capable of producing pellicles protects better than parental BCG against TB in mice, particularly at 6 months post-infection or upon reactivation from persistent infection (Pedroza-Roldán et al., in preparation). The use of this vaccine candidate in animal models such as Non-Human Primates (NHP), which more closely reproduce latent infection, should allow us to determine whether biofilms share some aspects of this clinically relevant infection stage.

In the aforementioned study, we did not use bacteria grown as surface pellicles to immunize mice, so for now we cannot ascertain whether the BCG vaccine candidate termed BCGΔBCG1419c shows better protection than BCG because it forms some sort of pellicle *in vivo* therefore presenting specific antigens not induced by regular BCG, or because deletion of the c-di-GMP phosphodiesterase *BCG1419c* gene render the bacteria with augmented levels of this second messenger, which has adjuvant properties (Chen et al., 2010).

New vaccine candidates could result from particular analyses around the biofilm mode of growth, by taking advantage of RNA sequencing and bioinformatics (to monitor gene expression differences during biofilm mode of growth *in vitro*), real time qPCR (to seek for correlation between expression of genes differentially transcribed during biofilm production with that obtained during *in vivo* infection), proteomics (to evaluate correlation between transcription differences and production of antigenic peptides and proteins relevant for biofilm mode of growth), immunology (to compare *in vivo* and/or *ex vivo* the cellular and humoral immune response mounted by immunized subjects/models, toward specific components relevant for biofilm production), and genomics (to determine whether putative “weak” biofilm producers used for vaccine production differ in sequences of genes required to control this phenotype). We should also consider comparing expression of molecules relevant for biofilm production with that already found under experimental conditions that have been demonstrated to occur during *in vivo* infection, such as hypoxia, reactive oxygen/nitrogen species stress, nutrient limitation and shift in carbon source utilization, in order to rule out utilization of antigens already under study or demonstrated to be poor vaccine candidates.

I suggest that we should further continue evaluating the relevance of the biofilm phenotype to produce vaccine candidates against TB, by following at least one of the next approaches: (1) genetically engineered, whole cell mycobacteria with enhanced capacity to produce biofilm and persist longer *in vivo* yet displaying a good (attenuated) safety profile, (2) through the use of specific components produced at different times during biofilm production, which I recommend be confirmed to be expressed during *in vivo* infection, or (3) via utilization of components exclusively or more abundantly produced in biofilms than in planktonic cells, and test them as subunit, perhaps booster vaccines candidates, or (4) particular components produced at different locations within the same biofilm, which in other words implies utilizing modern technologies to map transcriptional diversity depending on location within the biofilm. Assessment of all or some of these alternatives should shed light on how successful paying a critical re-evaluation of biofilms could be against this disease. These vaccine candidates, perhaps administered using the respiratory route (which has shown improved control of TB in NHP; Kaushal et al., 2015; White et al., 2015) when delivery and safety concerns have been solved, would likely result in better control of tuberculosis in the near future.

## Author contributions

MF conceived the idea and wrote the manuscript.

## Funding

No specific grant supported this work, although partial funding to start working on these ideas was provided by Fondo Sectorial de Investigación en Salud y Seguridad Social-CONACYT 86396 as well as internal funding from CIATEJ, A.C.

### Conflict of interest statement

Dr. Mario A. Flores-Valdez has filed for patents on the use of BCGΔBCG1419c as vaccine against tuberculosis.
